# Case Report: Can early full-course tocilizumab therapy reverse vascular stenosis in Takayasu arteritis?

**DOI:** 10.3389/fimmu.2025.1593770

**Published:** 2025-06-26

**Authors:** Congqi Hu, Lingjie Liu, Hui Xiao, Hongjun Zhao, Guangxing Chen, Yanli Xie

**Affiliations:** ^1^ Department of Rheumatology Nephrology, Baiyun Hospital The First Affiliated Hospital of Guangzhou University of Chinese Medicine, Guangzhou, Guangdong, China; ^2^ First Clinical Medical School, Guangzhou University of Chinese Medicine, Guangzhou, Guangdong, China; ^3^ Department of Rheumatology Nephrology, Suining County People’s Hospital, Shaoyang, Hunan, China; ^4^ Department of Rheumatology and Immunology, Xiangya Hospital, Central South University, Changsha, Hunan, China

**Keywords:** Takayasu arteritis, tocilizumab, TNFi, vascular stenosis and recanalization, cure

## Abstract

Takayasu arteritis (TAK) is an idiopathic systemic disease characterized by granulomatous inflammation of the aorta and its branches. TAK can cause multiple vascular injuries throughout the body, mainly arterial stenosis and aneurysms. In severe cases, it can even lead to fatal hemorrhage, infarction and other serious complications, posing a serious threat to the patient’s life and health. Few studies have shown that drug treatment can improve or reverse its vascular stenosis. This study describes a 19-year-old woman diagnosed with TAK who had multiple vascular stenosis at the time of the disease. In an early and timely manner as well as up to seven years after a full course of tolizumab, her vascular wall thickening improved and previously stenotic vessels were recanalized. We believe that early use of tocilizumab in patients with TAK can improve vascular lesions. To our knowledge, this study is the first case to find complete recanalization of stenotic vessels after the use of tocilizumab, and the pre- and post-test and examination data are complete. In addition, we summarized the cases of improvement of vascular lesions after tocilizumab treatment of TAK, and preliminarily compared the efficacy and safety of tocilizumab and TNFi in TAK. We speculate that early and adequate use of tocilizumab could reverse early inflammatory vessel wall thickening and stenosis, and we found, through further literature review, that its efficacy was comparable to that of TNF inhibitors

## Highlights

- A patient with Takayasu arteritis experienced revascularization of previously narrowed vessels following treatment with tocilizumab.
- Early use of tocilizumab in Takayasu arteritis may improve vascular stenosis.
- Equivalent efficacy of tocilizumab versus TNF inhibitors in the treatment of Takayasu arteritis.

## 1 Introduction

Takayasu arteritis (TAK) is a chronic disease characterized by granulomatous inflammation involving the aorta and its main branches, which can easily lead to arterial stenosis, occlusion or aneurysm formation, and clinical manifestations include limb claudication, pulselessness, organ damage, and even death (1, 2). Epidemiological surveys show that in Europe, the incidence of TAK is 0.4 to 3.4 per million per year, and it mainly affects young women (3, 4).

The pathogenesis of TAK is still unclear. Current studies have shown that its mechanism may be granulomatous inflammation of the vascular wall and abnormal immune response to injury, which promotes intimal hyperplasia, adventitial thickening and intramural angiogenesis, ultimately affecting vascular integrity and tissue perfusion (4). Vascular damage is mainly manifested as vascular stenosis and occlusion, which is prone to involve the cardiovascular system. Complications such as myocardial infarction, stroke, arterial rupture and arterial dissection are key factors affecting its prognosis and mortality. Current treatments for TAK include glucocorticoids, immunosuppressants and biological agents, all of which can improve vascular inflammation, but as far as we know, there is no strong evidence that drug therapy can reduce or reverse vascular stenosis. At present, clinically, the absence of any clinical features attributable to active disease, normalization of laboratory indicators, and cessation of abnormal progression of vascular imaging are defined as disease remission (3). For severe arterial stenosis causing coronary artery involvement, rapidly progressive tissue or organ infarction, or limb claudication that seriously affects activity, surgical treatment can be considered (5).

Currently, few studies have shown that drug therapy alone can cause TAK-induced vascular stenosis to regress or reverse, and the current management guidelines issued by the American College of Rheumatology/Vasculitis Foundation (ACR/VF) in 2021 recommend TNFi over tocilizumab in the treatment of TAK. However, this study describes a case in which vascular stenosis regressed after tocilizumab treatment. In addition, we also systematically reviewed the literature on the improvement or regression of vascular stenosis after TAK treatment, and compared and analyzed the efficacy and safety of TNFi and tocilizumab in TAK. We believe that early application of tocilizumab in adequate doses and courses is beneficial in improving vasculopathy in TAK and has comparable efficacy to TNF inhibitors.

## 2 Case report

A 19-year-old female was referred to the Department of Rheumatology and Immunology, Peking Union Medical College Hospital on January 27, 2016, for chest pain and fever. The main complaint was chest pain for 3 months and intermittent fever for 1 month.

Physical examination showed: blood pressure was 74/40 mmHg in the left upper limb and 82/44 mmHg in the right upper limb. There was tenderness in the 3rd and 4th rib joints on both sides, especially on the right side, and grade I-II vascular murmurs were present in the lower part of both clavicles and the right upper abdomen.

**Figure 1:**
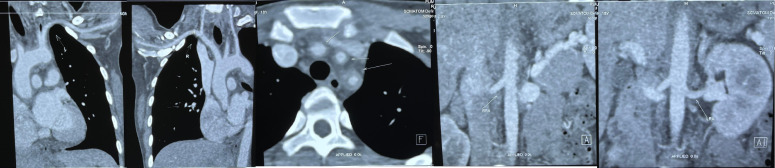
Pre-treatment Imaging. Aortic CTA changes before treatment: thickening of the walls of the ascending aorta, aortic arch, and descending aorta; sheath-like changes could also be seen at the root of each branch of the aortic arch, and mild to moderate narrowing of the lumens of the left common carotid artery, left subclavian artery, and brachiocephalic trunk. Abdominal aorta involvement was mild.

Laboratory tests: ESR>140mm/h, hs-CRP 136.28mg/L, Ferritin 493ng/mL, HB 90g/L. Examination tips: Aortic CTA: irregular circumferential thickening of the walls of the ascending aorta, aortic arch, and descending aorta; mild to moderate narrowing of the lumen of the left common carotid artery, left subclavian artery, and brachiocephalic trunk; and involvement of the abdominal aorta (**Figure 1**).

According to the 2022 ACR/EULAR classification criteria for TAK (classification criteria should not be used for diagnosis), the age at diagnosis is ≤60 years old, and the imaging evidence of vasculitis meets the admission conditions. The patient is female (1 point), has a vascular murmur (2 points), and has more than 3 affected arteries. (3 points), 6 points in total, the diagnosis of TAK is clear (1, 6). Treatment was initiated with prednisone acetate (40 mg po qd), aspirin (100 mg po qd), and leflunomide (20 mg po qd). However, during the one-year treatment, although the inflammatory indicators decreased, they only reached the target once. And with the prednisone tapered to 15mg, the patient developed new symptoms of pain in the neck and inner thighs. Considering that the TAK disease activity was not controlled, there was signs of relapse. After communicating with the patient, tocilizumab treatment was started (400mgVD, reduced to 320mgVD after 3 uses). After one treatment with tocilizumab, the patient’s clinical symptoms improved significantly, and ESR and hs-CRP dropped to normal. This regimen was maintained and the dose of prednisone acetate was gradually reduced to 7.5 mg/d. No new symptoms occurred during this period.

Tocilizumab was injected once every 5–6 weeks (7.2mg/kg) from January 2017 to February 2024, with a total of more than 50 doses, during which no disease recurred.

**Figure 2:**
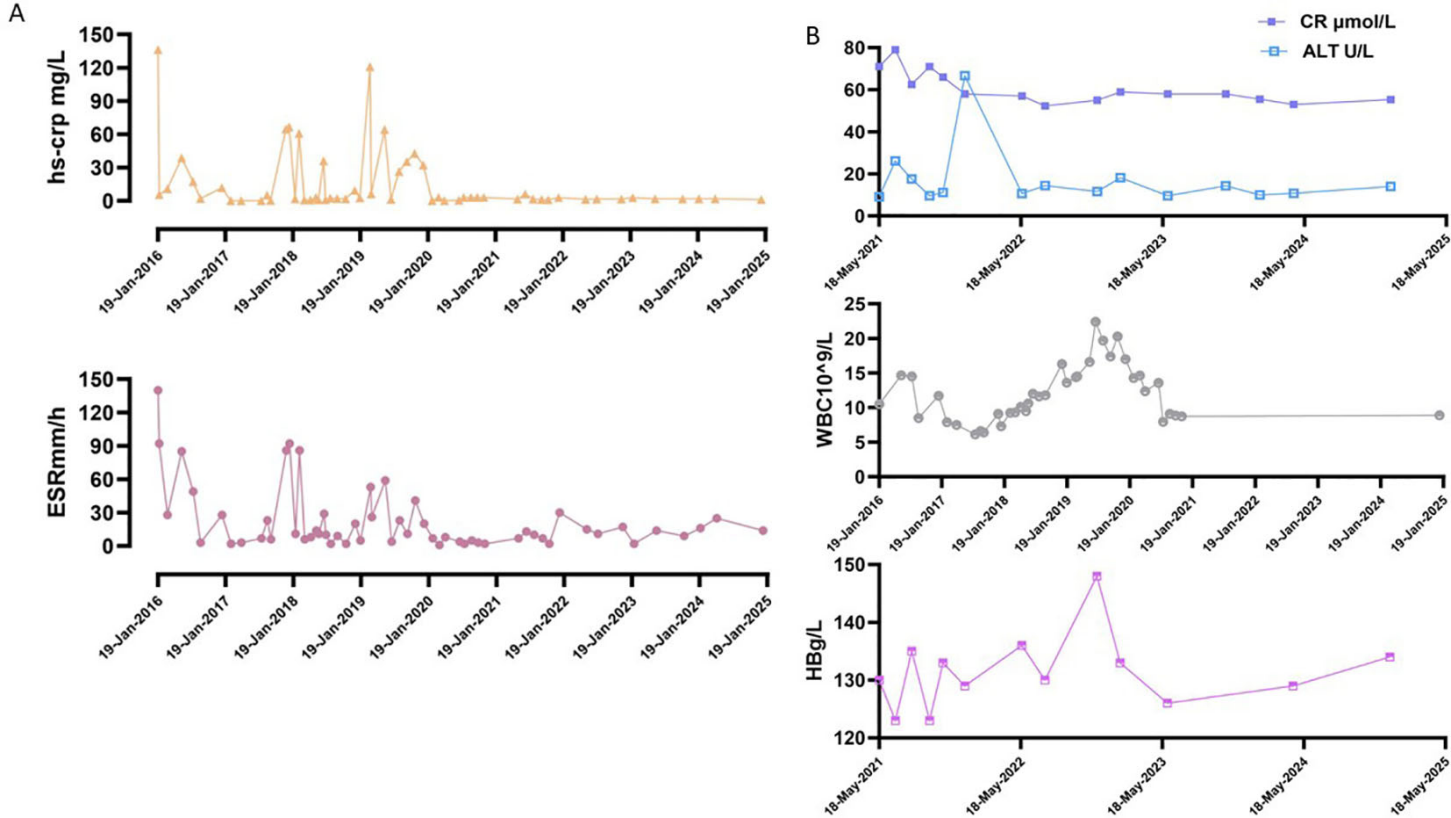
Summary of examination indicators during the treatment period. **(A)** Inflammation indicators change during treatment. Normal reference value: ESR, 0~20mm/h; hs-CRP, 0~3mg/L. **(B)** The data of some indices during the treatment period, including WBC, HB, CR and ALT. Normal reference values: WBC, 4 - 10×10^9^/L; HB, 110–150 g/L; CR, 41 - 111 μmol/L; ALT, 7.0 - 40.0 U/L.

ESR and hs-CRP were monitored during tocilizumab treatment. At the beginning of treatment, ESR>140mm/h, hs-CRP136.28mg/L, and inflammatory indicators improved after treatment. The two increases in inflammatory indicators during the period were due to the absence of injections for 11 weeks after the 8th injection and the absence of injections for nearly 8 weeks after the 9th injection. Thereafter, inflammatory indicators could be maintained normal when tocilizumab was injected at intervals of 5–6 weeks (**Figure 2**). After tocilizumab treatment, the patient’s clinical symptoms and laboratory tests were well controlled. Tocilizumab did not cause any side effects, including hepatotoxicity, renal toxicity, and blood system side effects (**Figure 2**).

**Figure 3:**
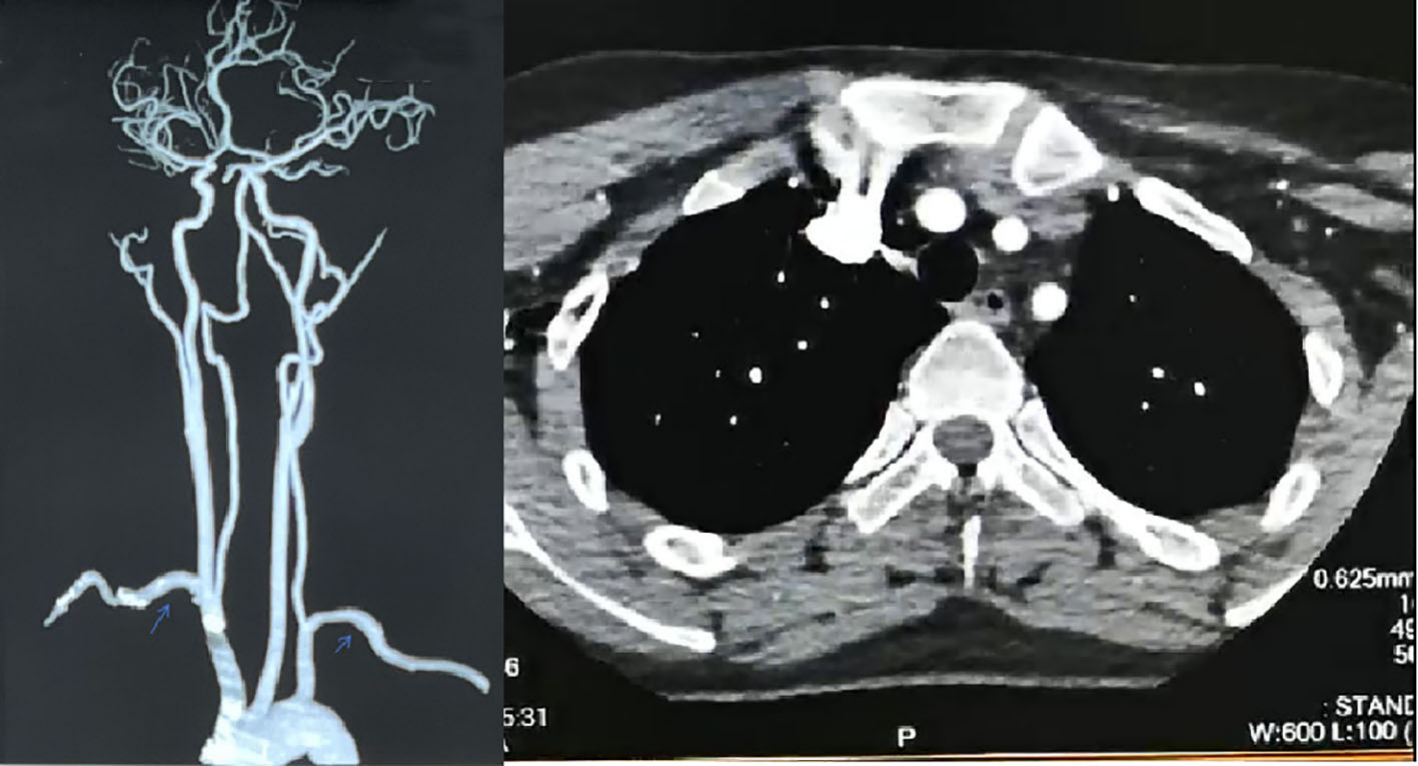
Post-treatment images. After treatment, full-length aortic CTA+ internal carotid artery imaging CTA: The wall of the aortic arch was slightly thickened, about 2mm, and no significant narrowing was observed in the lumen. There were no obvious filling defects, localized strictures, tumor-like dilatation or malformed vascular masses in the ascending aorta and main branches of the aorta (brachiocephalic trunk, left common carotid artery, left subclavian artery). There were no obvious signs of stenosis or dilatation in bilateral common carotid arteries.

In February 2024, she was admitted to Xiangya Hospital of Central South University. The full-length aortic imaging CTA, CT brain plain scan + intracranial artery + carotid artery imaging CTA showed: 1. Thickening of the aortic arch wall 2. No obvious abnormalities were found in CT brain plain scan + intracranial artery + carotid artery imaging CTA. Compared with the aortic CTA in January 2016, the ascending aorta, the main branches of the aorta (brachiocephalic trunk, left common carotid artery, left subclavian artery), the abdominal aorta and its branches did not show obvious filling defects, localized stenosis, tumor-like dilatation and abnormal vascular mass. No obvious signs of stenosis and dilatation were found in the bilateral common carotid arteries (**Figure 3**). PET-CT results: No obvious localized abnormal glucose metabolism was found in the ascending aorta, aortic arch, descending aorta and its branches: indicating that there is no obvious active large artery inflammation, and follow-up reexamination is recommended. Examination after nearly one-year follow-up indicated that the patient had normal inflammatory markers. There was no recurrence of clinical symptoms, and no adverse reactions occurred.

This article has obtained the approval of the Medical Ethics Committee of Xiangya Hospital, Central South University, with the batch number 201212074, and both the data and the imaging reports in the article have obtained the informed consent of the patients.

## 3 Literature review

In addition to the case reported in this study, all available previous studies were retrieved by performing a systematic review of cases with improvement in TAK with tocilizumab. Studies published in international journals included in the PubMed, Embase, and Cochrane database from January 2000 to January 2024 were analyzed. Patients diagnosed with TAK and treated with tocilizumab were included in the studies. Studies published in English were selected, and additional cross-checks were performed on the references cited therein. As a search strategy, a combination of the following terms was used: “Takayasu”, “Takayasu arteritis”, “anti-IL6”, “tocilizumab”, “vasculopathy”, “treatment” or “treatment improvement”, and 6 studies were retrieved. A total of 6 cases of TAK successfully treated with tocilizumab with complete data were reported (7–11) (**Table 1**).

**Table 1:** A summary of cases of patients with Takayasu arteritis showing improvement in their conditions after treatment with tocilizumab.

Author	Date	Sex, (age, years)	Disease	Initial therapeutic protocol	Disease course prior to the use of tocilizumab/month	Adjusted therapeutic protocol	Pre-treatment examination results	Laboratory indicators	After treatment, vascular condition	Test results	Clinical symptoms	Adverse reactions
Norihiro Nishimoto (7)	2008	female (15)	TAK and UC	Corticosteroidscyc, losporin A, cyclophosphamide, azathioprin, mycophenolate, methotrexate	61.9	Tocilizumab, prednisolone	Marked thickening of the wall of the ascending aorta, the three branches of the aortic arch (right brachiocephalic artery, left common carotid artery, and left subclavian artery), and the descending aorta, as well as stenosis of the left subclavian artery. Suspected stenosis of the right common carotid artery.	ESR, CRP, IL-1, IL-6, TNF	Enhanced CT analysis revealed a reduction in the thickening of the wall of the ascending and descending aorta	control normalized	Episodes of syncope disappeared after the start of tocilizumab treatment, and arterial pulses became easily palpable,	Not found
YokokawaT (8)	2019	female (18)	TAK	prednisolone	3	Tocilizumab, Methotrexate and prednisolone	Coronary angiography showed severe stenosis in the ostium of both the left main trunk and the right coronary artery, % diameter stenosis of the ostial stenosis was 95.0% in the left main trunk and 87.2% in the right coronary artery	ESR, CRP, SAA	diameter stenosis was 86.7% in the left main trunk and 72.6% in the right coronary artery	No significant increase	Patient did not have chest oppression or ST-segment depression after the immunosuppressive treatment. She had no cardiac events for 6 months after discharge.	Not found
Bravo Mancheño B (9)	2012	female (3)	TAK	prednisone, MTX, Etanercept, infliximab, CYC, acetylsalicylic acid, clopidogrel, MMF	29.4	implantation of a stent in the brachiocephalic branch, PCTA(a significant restenosis), TCZ, corticosteroids, Mycophenolate Mofetil	significant thickening of the aortic-arch wall, with severe stenosis of the brachiocephalic branch and the left subclavian artery, Both common carotid arteries were completely occluded, with retrograde flow in the internal carotid arteries originating from the vertebrobasilar system	ESR, CRP, IL-6, TNF – α, SAA, PLT	the stent remains permeable with moderate stenosis (gradient 50 mm Hg), and the thickness of the aortic arch wall has decreased (3 mm compared with 5 mm previously). Both common carotid arteries, which were completely occluded, received partial recanalization, with anterograde flow in the internal carotids and the images of brain ischemia have improved,	remained within the normal range	have palpable pulses, with blood pressure readings in the normal range.	Not found
Decker P (10)	2018	female (20)	TAK	Hormones, azathioprine, methotrexate	27.3	TCZ	The walls of the aortic arch, right brachiocephalic trunk, and right common carotid artery are thickened, the hypoechoic right carotid artery is thickened, and the right subclavian artery is thickened with stenosis.	CRP	Thickening of the wall of the right common carotid artery disappeared	Decreased to normal	Neck pain disappeared	Not found
Decker P (10)	2018	female (13)	TAK	Hormones, methotrexate, infliximab	25.4	tocilizumab, hormones	Moderate FDG uptake in the aortic arch, descending aorta, left internal carotid artery, and left subclavian artery	CRP	PET-CT was measured in July 2016 and no FDG uptake was observed.	ease	Clinical symptoms improved	Not found
Risse J (11)	2016	male (19)	TAK	Low molecular weight heparin versus intravenous corticosteroids, aspirin, and beta-blockers	0.5	Tocilizumab, surgical treatment	PET-CT showed hypermetabolism in the ascending thoracic aorta, proximal part of the aortic arch, and right external iliac artery. Left ventricular ejection fraction 18%	CRP, BNP	After 3 months, PET-CT showed no active vasculitis and the heart function was restored to almost normal. After 18 months, the disease was no longer active.	Down to normal	Most clinical manifestations disappeared	Not found

TAK, Takayasu arteritis; UC, Ulcerative colitis; TCZ, Tocilizumab; CT, Computed Tomography; FDG, Fluorodeoxyglucose Positron Emission Tomography; PET-CT, Positron Emission Tomography/Computed Tomography; PCTA, Percutaneous Transluminal Coronary Angioplasty; ESR: Erythrocyte Sedimentation Rate; CRP, C-Reactive Protein; IL-1, Interleukin-1; IL-6, Interleukin-6; SAA, Serum Amyloid A; TNF, Tumor Necrosis Factor; BNP, Brain Natriuretic Peptide; PLT, Platelet.

The cases included 5 TAK patients and 1 TAK combined with ulcerative colitis (UC), including 5 females and 1 male. All cases had been treated with glucocorticoids before using tocilizumab. The average time of starting tocilizumab in case 1 and cases 3–5 was the 36th month of the disease course. All cases were treated with tocilizumab after poor results of hormone, immunosuppressant or non-tocilizumab biological agents. Case 2 used tocilizumab 3 months after onset. Case 6 was a severe TAK patient, and tocilizumab was used as a preoperative treatment to quickly control disease activity.

Cases 3 and 6 were treated with combined surgical treatment. Case 3 underwent brachiocephalic branch stenting, and restenosis was found in the postoperative PCTA examination. After the use of tocilizumab, the imaging improvement was found in the follow-up examination. Case 6 underwent aortic mesothelial and aortohepatic bypass surgery, and tocilizumab was continued after the operation until the disease was relieved.

Case 1 and Case 4 added tocilizumab to the original treatment plan to achieve reduction in vessel wall thickening. Case 2 used tocilizumab and combined with hormone pulse therapy, and reexamination found that arterial stenosis had subsided. Case 3 had poor efficacy with etanercept, and after headaches occurred with infliximab, tocilizumab was used. Reexamination found that some vascular wall thickening had improved and arterial occlusions had partially recanalized. Case 5 did not improve with infliximab, and reexamination of PET-CT after tocilizumab showed radiological relief without high uptake. Case 6 continued to use tocilizumab after surgery, and PET-CT did not find active vasculitis, and the disease remission was achieved. Case 1, Cases 3–6 all considered to have achieved hormone reduction, decreased inflammatory indicators, improved examination results, and relief of clinical symptoms due to the efficacy of tocilizumab. The better improvement in Case 2 may be due to the combined effects of tocilizumab, hormone pulses, and immunosuppressants. No adverse reactions or recurrence were observed in the 6 patients using tocilizumab.

## 4 Discussion

TAK is a rare chronic idiopathic granulomatous large vessel inflammation that can easily cause vascular lesions and lead to organ ischemia. Disease remission is defined as the cessation of abnormal progression of vascular imaging in terms of vascular lesions. Currently, no high-quality studies have shown that drugs can improve vascular stenosis (only some case reports have found that patients’ vascular lesions have improved after using glucocorticoids or immunosuppressants, but most studies have problems such as incomplete data). In 2008, Norihiro Nishimoto reported that the thickening of the ascending and descending aorta walls was reduced after tocilizumab treatment of TAK, and there were complete medication records and test and examination data. This article included it as case 1 in the literature analysis (7). Cases 2, 3, and 4 also found that tocilizumab treatment improved the patient’s vascular stenosis and reduced wall thickening. The vascular stenosis of the case in this study was completely recanalized after tocilizumab treatment, and the treatment lasted for up to 6 years. During this period, regular follow-up and complete test and examination data were obtained, suggesting that early use of tocilizumab may reverse TAK vascular stenosis.

Combined with the pathogenesis of TAK, it is currently believed that under the influence of genetic, environmental and other factors, immune intolerance and the cascade reaction of proinflammatory mediators will lead to progressive tissue damage. Stimulated dendritic cells release various effector molecules and cytokines through proinflammatory cells such as macrophages and T cells to drive vascular inflammation. Continuous vascular inflammation and attempted remodeling lead to neovascularization, arterial wall edema, and vascular wall damage, including intimal hyperplasia and fibrosis, with clinical manifestations of arterial stenosis, occlusion, and aneurysm formation (4, 12).

IL-6 can participate in vascular inflammation and fibrosis through multiple pathways. It induces mitochondrial phosphorylation of STAT3 (Tyr705) to prevent MFN2 proteasome degradation, promotes aging-related mitochondrial dysfunction, and leads to VSMCs aging and vascular inflammation (13); promotes T cells to differentiate into IL-17-producing Th17 cells, driving vascular inflammation (14–16). Combined with the analysis of serum cytokine profiles during the treatment of cases 1 and 3, IL-6 may indirectly affect the production of TNF-α and participate in granuloma formation (7, 9); it activates Jak1 and Jak2/stat3 pathways to initiate autophagy, participates in the process of vascular fibrosis (17), and leads to vascular stenosis.

Tocilizumab is a humanized anti-IL-6R antibody that blocks the IL-6R signaling cascade by blocking the binding of IL-6 to IL-6R (18). Studies have shown that the detection of IL-6 during tocilizumab treatment can reflect disease activity, suggesting that tocilizumab can inhibit IL-6 (19), improve or reverse arterial wall edema and inflammatory arterial stenosis through multiple pathways and mechanisms, and prevent myofibroblast proliferation from driving intimal fibrosis and leading to non-inflammatory vascular stenosis. This may partly explain the improvement of vascular stenosis in some patients reported in certain cases (20), and may confirm the efficacy of tocilizumab in treating TAK (21).

In a randomized, double-blind study of tocilizumab versus placebo conducted in Japan, although the primary endpoint (time to recurrence) did not reach statistical significance between the treatment groups, there was a favorable trend (22). Subsequent long-term efficacy and safety studies have shown that the use of tocilizumab helps stabilize or improve imaging assessments, reduce glucocorticoid doses, and has no safety issues (23). Some meta-analyses and retrospective studies have also shown that tocilizumab helps patients improve radiological outcomes and overall efficacy, and is generally safe, which is consistent with previous clinical research results (24–26).

However, the ACR guidelines updated in 2021 consider that TNFi has more clinical experience and data in TAK, and prefer TNFi to treat TAK, while TCZ can be considered for use (2). In an open-label study comparing the efficacy and safety of adalimumab (ADA) versus tocilizumab (TCZ) in patients with active and severe Takayasu’s arteritis, it was shown that ADA combined with glucocorticoids (GCs) and methotrexate (MTX) may be more effective than TCZ combined with GCs and MTX. Nevertheless, the article’s conclusions were limited by a small sample size and short observation period (27).

Furthermore several clinical studies have shown that the clinical response, angiographic stability, and safety of tocilizumab or TNFi in the treatment of TAK are similar (28–30). A meta-analysis of six controlled observational studies also found that the clinical remission rate [risk ratio (RR) tocilizumab vs TNFi 1.03, 95% CI (0.91-1.17)], angiographic stability rate (RR 1.00, 95% CI 0.72-1.40), or adverse event rate (RR 0.84, 95% CI 0.54-1.31) of tocilizumab or TNFi were similar (28). Studies evaluating the long-term outcomes of biological targeted therapy in TAK also showed that there was no significant difference in the efficacy and safety of TNFi antagonists and tocilizumab, preliminary experience suggests that tocilizumab may be another option for refractory TAK (31).

Case 3 and Case 5 in this article achieved clinical improvement after initiating tocilizumab after poor efficacy of TNFi, and the cases reported in this article and Case 2 showed good improvement in vascular stenosis. Case 1, Cases 3–5 all used hormones and immunosuppressants for a long time, and even started tocilizumab treatment after TNFi was ineffective. Although the average time to initiate tocilizumab was the 36th month of the course of the disease, radiological improvement or stable efficacy was achieved. It is worth mentioning that in this case and case 2, early initiation of tocilizumab to control vascular inflammation resulted in improvement in vascular stenosis and even complete recanalization. Therefore, combined with current research and the cases summarized in this article, we speculate that early initiation of tocilizumab in the treatment of refractory TAK can relieve vascular inflammation and fibrosis as soon as possible, improve vascular stenosis, and have comparable efficacy to TNF inhibitors, but high-quality research is still needed to further confirm it.

Furthermore, to our knowledge, this study represents the first documented case of complete recanalization of vascular stenosis in TAK patients achieved through prolonged tocilizumab therapy, with comparative aortic CTA imaging confirming full vascular restoration both pre- and post-treatment. This patient used tocilizumab for 6 years, with sufficient clinical index data and complete imaging data. The longest time for evaluating the long-term treatment results of tocilizumab in the published articles is 3 years (31). We speculate that long-term cumulative doses may also play a role in the recanalization of stenotic vessels. After the whole-body CTA and PET-CT of this patient were reviewed on February 2024, it was found that the previous vascular stenosis was completely recanalized and there was no sign of vasculitis. Therefore, tocilizumab was discontinued and low-dose prednisone acetate (2.5 mg po qd) combined with tofacitinib (5 mg po bid) was used for treatment. The patient has been followed up for 8 months and there is no sign of recurrence.

However, this study also has certain limitations. 1. This study is a systematic review based on case reports, and the level of evidence is not high. We hope that there will be high-quality clinical studies to further confirm this. 2. The follow-up period is short. There is a possibility of recurrence of TAK after discontinuation of tocilizumab. Currently, this patient has not relapsed after 8 months of follow-up, but the follow-up time is short. We will continue to pay attention to the patient’s follow-up. 3. There are cases with good responses in the included literature, but there are also cases with poor effects, which may be caused by the complexity of refractory TAK, which also suggests the importance of regular imaging review in TAK (32).

## 5 Conclusion

By comparing and analyzing various studies, we found that drug therapy is effective for TAK, but it is rare to achieve recanalization of vascular stenosis by drug therapy alone. This study suggests that early and adequate treatment with tocilizumab in refractory TAK can reverse early inflammatory vascular wall thickening and vascular stenosis. However, the controversy over the use of tocilizumab and TNFi shown in guidelines and research results also needs to attract the attention of clinicians. The present study further conducted a systematic literature review and found that current evidence suggests that tocilizumab and adalimumab are similarly effective in the treatment of aortitis, and this issue still needs to be further confirmed by high-quality clinical studies.

## Data Availability

The original contributions presented in the study are included in the article/supplementary material. Further inquiries can be directed to the corresponding authors.
